# A Shaking-Culture Method for Generating Bone Marrow Derived Mesenchymal Stromal/Stem Cell-Spheroids With Enhanced Multipotency *in vitro*

**DOI:** 10.3389/fbioe.2020.590332

**Published:** 2020-10-20

**Authors:** Kunimichi Niibe, Yumi Ohori-Morita, Maolin Zhang, Yo Mabuchi, Yumi Matsuzaki, Hiroshi Egusa

**Affiliations:** ^1^Division of Molecular and Regenerative Prosthodontics, Tohoku University Graduate School of Dentistry, Sendai, Japan; ^2^Department of Biochemistry and Biophysics, Graduate School of Medical and Dental Sciences, Tokyo Medical and Dental University, Tokyo, Japan; ^3^Department of Life Science, Faculty of Medicine, Shimane University, Matsue, Japan; ^4^Center for Advanced Stem Cell and Regenerative Research, Tohoku University Graduate School of Dentistry, Sendai, Japan

**Keywords:** mesenchymal stem cell, multipotency, shaking-culture, stem cell pool, spheroid

## Abstract

Mesenchymal stromal/stem cells (MSCs), which generally expand into adherent monolayers, readily lose their proliferative and multilineage potential following repeated passages. Floating culture systems can be used to generate MSC spheroids, which are expected to overcome limitations associated with conventional adherent cultures while facilitating scaffold-free cell transplantation. However, the phenotypic characteristics of spheroids after long-term culture are unknown. In addition, regenerative therapies require new culture systems to maintain their undifferentiated state. In this study, we established a novel culture method employing three-dimensional (3D) “shaking” to generate MSC spheroids using bone marrow derived MSCs. Floating 3D cultures of mouse or human MSCs formed spheroids after shaking (85–95 rpm), within 1 month. These spheroids maintained their osteogenic-, adipogenic-, and chondrogenic-differentiation capacity. The adipogenic-differentiation capacity of adherent cultured mouse and human MSCs, which is lost following several passages, was remarkedly restored by shaking-culture. Notably, human MSC spheroids exhibited a renewable “undifferentiated MSC-pool” property, wherein undifferentiated MSCs grew from spheroids seeded repeatedly on a plastic culture dish. These data suggest that the shaking-culture method maintains and restores multipotency that is lost following monolayer expansion and thereby shows potential as a promising strategy for regenerative therapies with mesenchymal tissues.

## Introduction

Bone marrow, which contains mesenchymal stromal/stem cells (MSCs), is a useful cell source that can be applied for regenerative therapies (Prockop, 1997; Pittenger et al., 1999; Egusa et al., 2012; Shammaa et al., 2020). The clinical application of bone marrow derived MSCs (BM-MSCs) is widespread, due to their proliferative ability and multipotent differentiation potential. However, a critical issue associated with the use of BM-MSCs is that, under adherent monolayer culture conditions, these cells eventually lose their stemness properties following repeated passages during culture expansion (Bonab et al., 2006; Stolzing et al., 2006; Wagner et al., 2008; Bork et al., 2010; Gu et al., 2016). It has been suggested that supplementing the culture medium with fibroblast growth factor (FGF)-2 (Tsutsumi et al., 2001; Cheng et al., 2020; Xu et al., 2020) and epidermal growth factor (EGF) (Tamama et al., 2006; Xu et al., 2020) can enable large-scale expansion of MSC cultures. However, these methods are limited as such supplements cannot sufficiently maintain the cellular differentiation potential in long-term culture (Gharibi and Hughes, 2012; Cheng et al., 2020).

Three-dimensional (3D) cultures provide a more suitable environment for cells that rarely grow in two-dimensional (2D) monolayers under physiological conditions. Multicellular aggregates in 3D cultures, termed “spheroids,” have been widely used in cancer research (Nath and Devi, 2016), stem cell biology (Cesarz and Tamama, 2016), and tissue engineering (Han et al., 2019). Recently, several techniques, such as the hanging-drop method (Bartosh et al., 2010), chitosan membranes (Huang et al., 2011), forced aggregation in microwells (Baraniak and McDevitt, 2012), low-binding plates (Redondo-Castro et al., 2018), and magnetic levitation (Lewis et al., 2017) have been developed to generate MSC spheroids (mesenspheres). MSC spheroids reportedly address many limitations associated with conventional adherent MSC cultures, facilitating scaffold-free cell transplantation with desirable phenotypes (such as enhanced anti-inflammatory effects and survival rates following transplantation), and showing improved MSC stemness (Cesarz and Tamama, 2016). Previously, floating dynamic culture systems for culturing freshly isolated MSCs were reported. MSCs were cultured in the rotation platform (65 rpm, 2 days) (Hildebrandt et al., 2011) or the shaker flask (80 rpm, 7 days) (Alimperti et al., 2014) to generate MSC spheroids. These short-term cultured MSC spheroids kept osteogenic or tri-lineage (osteogenic, adipogenic, and chondrogenic) differentiation potential. However, the phenotypic fate of spheroids following long-term culture remains unknown. In addition, regenerative therapies await a simpler 3D culture platform, which enables the maintenance of stem cell stemness.

Another potential issue associated with the use of human BM-MSCs (hBM-MSCs) is that their therapeutic success rate varies among patients due, in part, to the heterogeneity of BM-MSC populations (Toyoda et al., 2007; Morikawa et al., 2009a). In 2006, the International Society for Cellular Therapy (Dominici et al., 2006) defined hBM-MSCs as cells (1) with the capacity to attach to plastic culture dishes, (2) that express specific surface antigens, and (3) with multipotent differentiation potential toward mesenchymal lineage cells. However, these criteria were not definitive, and a lack of specific cell-surface markers to define MSCs has hindered the understanding of molecular aspects associated with the cellular characteristics of these cells (Niibe et al., 2017). In contrast, specific markers for hematopoietic stem cells (HSCs) and hematopoietic lineages are well established (Morrison et al., 1995; Seita and Weissman, 2010). HSCs can be isolated and analyzed by flow cytometry. HSC transplantation therapy has achieved remarkable and reproducible results (Copelan, 2006). However, it is essential to identify specific BM-MSC markers to develop standardized BM-MSC-based basic research and treatment protocols. Recently, combinations of cell-surface BM-MSC markers have been identified, including mouse platelet-derived growth factor receptor α (PDGFRα: also known as CD140a) and stem cell antigen-1 (Sca-1) in mice (Morikawa et al., 2009b), as well as human low-affinity nerve growth factor receptor (CD271), THY-1 (CD90), and vascular cell-adhesion protein-1 (VCAM-1/CD106) (Mabuchi et al., 2013). These marker combinations enable the prospective isolation of highly enriched BM-MSC populations (Morikawa et al., 2009b; Mabuchi et al., 2013), which shows the unique MSC characteristics of neural crest stem cell (NCSC) phenotypes (Morikawa et al., 2009b; Niibe et al., 2011b). Thus, enriched MSC studies more accurately recapitulate the phenotypic details of MSCs compared to whole bone marrow transplantation studies (Niibe et al., 2016). This prompted us to apply the unique and homogeneous properties of a highly enriched MSC population to fabricate spheroids.

Here, we present a simple 3D shaking-culture method that generates unique MSC spheroids, ensures the long-term maintenance of their multilineage potential, and restores multipotency that was lost following monolayer expansion of both mBM-MSCs and hBM-MSCs. In addition, spheroids from highly enriched hBM-MSC populations showed a renewable “undifferentiated MSC-pool” property, wherein undifferentiated MSCs grew from spheroids seeded on an adherent culture dish, following which each attached spheroid could then be removed and re-seeded several times to provide additional cultures of outgrown MSCs. Thus, this novel spheroid-culture method showed potential as a promising strategy for regenerating mesenchymal tissues.

## Materials and Methods

### Adherent MSC Culture

Freshly isolated mouse BM-MSCs (mBM-MSCs) from the bone marrow of C57BL/6 mice (Morikawa et al., 2009a; Houlihan et al., 2012) or commercially available mouse BM-MSCs (MUBMX-01001, Cyagen, Santa Clara, CA, United States) were used in this study. MUBMX-01001 MSCs were isolated from the bone marrow of C57BL/6 mice, and the cells at passage 6 were provided from the company. Mouse MSCs were maintained in MEM-α + GlutaMAX-I (Gibco BRL, Grand Island, NY, United States) supplemented with 10% fetal bovine serum (FBS; Hyclone: GE healthcare, Chicago, IL, United States), 1% penicillin-streptomycin solution (P/S; Wako, Osaka, Osaka, Japan), 10 mM HEPES (Dojindo Molecular Technologies, Inc., Kamimashiki, Kumamoto, Japan), and 20 ng/mL FGF-2 (Wako).

The mouse BM-MSCs enrichment method was described previously (Morikawa et al., 2009a; Houlihan et al., 2012). PE-conjugated CD45 (30-F11: eBisoscience), TER119 (TER-119: eBioscience), APC-conjugated PDGFRα (APA5: eBioscience), and FITC-conjugated Sca-1 (Ly6A/E: eBioscience) were used for the flow cytometry sorting, which was performed on Aria III flow cytometer (BD Biosciences, Franklin Lakes, NJ, United States). Propidium iodide (PI) fluorescence was measured, and a live cell gate was defined, which excluded cells positive for PI. Additional gates selected cells positive for PDGFRα and Sca-1 and negative for CD45 and TER119.

PI^–^/CD90^+^/CD271^+^ hBM-MSCs were sorted from the bone marrow of an 18-year-old male (product #2M-125C, lot #080514A; Lonza, Valais, Switzerland). Enriched hBM-MSCs were maintained in DMEM (Gibco) containing 20% FBS (Hyclone; GE Healthcare), 1% P/S (Wako), 10 mM HEPES (Dojindo Molecular Technologies, Inc.), and 20 ng/ml FGF-2 (Wako).

mBM-MSC and hBM-MSCs were cultured and incubated at 37°C with 5% CO_2_ at a density of 1 × 10^6^ cells/well in 10-cm dishes for 3 or 4 days. Once the cells reached 80% confluency, sequential passaging was performed until the cell-expansion limit was reached. Cells were washed with PBS without Mg^2+^ and Ca^2+^ [PBS(-)]. Cells were passaged with 0.25 w/v% trypsin-1 mol/L EDTA 4 Na Solution (Wako) added to the culture dish, and incubated at 37°C for 3 min. Next, trypsinization was neutralized by adding an equal volume of maintenance medium. Cells were pelleted by centrifugation at 280 × *g* for 5 min at 4°C, and re-seeded at 1 × 10^5^ cells/mL in a fresh dish.

### MSCs Shaking-Culture

mBM-MSCs were seeded at 5 × 10^4^ cells/mL (total: 1 × 10^6^ cells/20 mL) in 125-mL Erlenmeyer flasks (product #431405, Corning, Corning, NY, United States) with MSC adherence-maintenance medium, comprised of MEM-α + GlutaMAX-I (Gibco) containing 10% FBS (Hyclone; GE Healthcare), 1% P/S (Wako), 10 mM HEPES (Dojindo Molecular Technologies, Inc.), and 20 ng/mL FGF-2 (Wako). hBM-MSCs were seeded at 5 × 10^4^ cells/mL (total: 1 × 10^6^ cells/20 mL) or 5 × 10^5^ cells/mL (1 × 10^7^ cells/20 mL) in basic adherence-maintenance medium. The cells were cultured in a bio-shaker at 37°C with 5% CO_2_, a rotation speed of 85–95 rpm, and an amplitude of 40 mm (BR-40LF: TAITEC, Koshigaya, Saitama, Japan). Spheroids were transferred to a 50 mL centrifuge tube with culture medium, centrifuged at 1200 rpm for 5 min, and the supernatant was gently removed. Subsequently, half of the medium was renewed every 3–4 days.

### *In vitro*-Differentiation Assay

For osteogenic and adipogenic-differentiation, adherent MSCs were cultured in osteogenic or adipogenic induction medium (Lonza), respectively, with twice weekly medium changes. After 21 days differentiation into osteoblasts was confirmed via alkaline phosphatase (ALP)-based enzymatic staining (Vector Laboratories, Burlingame, CA, United States) and Alizarin red S staining (Sigma-Aldrich, St. Louis, MO, United States). After 21 days differentiation into adipocytes was confirmed via Oil red O (Muto Pure Chemicals, Bunkyo, Tokyo, Japan) staining, respectively (Morikawa et al., 2009b; Houlihan et al., 2012).

To induce chondrogenic-differentiation, cultured cells or spheres were transferred into 15-mL tubes containing chondrogenic-induction medium (Lonza) with 10 ng/mL transforming growth factor-β3 (R&D Systems, Minneapolis, MN, United States) and 500 ng/mL bone morphogenetic protein-6 (R&D Systems). The medium was changed twice weekly. After 21 days, chondrogenic-differentiation was confirmed via staining with toluidine blue (Wako) (Morikawa et al., 2009b; Houlihan et al., 2012).

### Neural Induction

To obtain neuronal spheroids, hBM-MSCs were cultured in a low-attachment dish (Corning) with serum-free sphere-forming medium comprising advanced DMEM/F-12 (1:1) (Gibco) supplemented with 0.5 mM l-glutamine, N2 supplement (Gibco), 20 ng/mL recombinant human EGF (Wako), 20 ng/mL human FGF-2 (Wako), and 2% B27 (Gibco). Following the formation of spheroids, neuronal spheroids obtained using low-attachment dishes or MSC spheroids obtained under shaking-culture conditions were seeded on fibronectin (Wako)-coated 6-well chambers (Matsunami Glass Ind. Inc., Kishiwada, Osaka, Japan) using induction medium comprising advanced DMEM/F12 (1:1) (Gibco) supplemented with 10% FBS (Hyclone, GE Healthcare), N2 supplement (Gibco), 1% P/S (Wako), and 10 mM HEPES (Dojindo Molecular Technologies, Inc.) (Morikawa et al., 2009b; Choi et al., 2017). The medium was changed every 3–4 days until day 10.

### Flow Cytometry Analysis

Flow cytometric analysis of MSCs was modified for the enrichment of mBM-MSCs (Morikawa et al., 2009b; Houlihan et al., 2012). Spheroids were re-attached and cultured for 3 days on a 10-cm dish. Monolayer MSCs and cells migrated from spheroids were washed with PBS (−), and cell dissociation buffer (enzyme-free, PBS; Gibco) was added to the culture dish and incubated at 37°C for 3 min. Then, the cells were neutralized by adding an equal volume of PBS (−). The cells were then pelleted by centrifugation at 280 × *g* for 5 min at 4°C. APC-conjugated PDGFRα (APA5, eBioscience, Santa Clara, CA, United States) and FITC-conjugated Sca-1 (Ly6A/E, eBioscience) were used for analyzing mouse MSCs. Flow cytometric analysis was performed using an Aria III flow cytometer (BD Biosciences). FITC-conjugated Thy-1 (CD90, BioLegend, San Diego, CA, United States) and APC-conjugated VCAM-1 (CD106, BioLegend) were used to analyze human samples. PI fluorescence was measured, and a live cell gate was defined by cells that excluded PI.

### Immunohistochemical Staining

Differentiated neuronal cells were fixed with PBS containing 4% paraformaldehyde, rinsed with PBS (−), and pretreated with PBS containing 0.3% Triton X-100 for 5 min at room temperature. After blocking the cells in tris-NaCl-blocking buffer for 30 min at room temperature, the cells were incubated overnight at 4°C with a primary anti-βIII-tubulin antibody (Abcam, Cambridge, United Kingdom). After washing with PBS, the cells were incubated for 1 h at room temperature with Alexa Fluor 488-conjugated anti-rabbit IgG H&L (Abcam) as the secondary antibody (Morikawa et al., 2009b). After washing with PBS, the samples were mounted and observed under a universal fluorescence microscope (LSM780; Zeiss, Oberkochen, Germany).

### H&E Staining

BM-MSC spheroids were fixed in freshly prepared PBS (−) containing 4% paraformaldehyde (pH 7.4) for 1 h and embedded in paraffin, using standard histological procedures. The spheroid blocks were cut into 8-μm thick sections and mounted on glass slides. For H&E staining, slides were deparaffinized with xylene and re-hydrated using an alcohol gradient of absolute alcohol, 95% alcohol, and 70% alcohol. The slides were then washed in distilled water and stained in hematoxylin solution (Muto Pure Chemicals) for 5 min. The slides were washed in running tap water for 5 min and counterstained in eosin Y solution (Muto Pure Chemicals) for 1 min. Stained slides were dehydrated using 70% alcohol, 95% alcohol, and 100% alcohol, and cleared in xylene twice for 5 min. The slides were then mounted in malinol (Muto Pure Chemicals).

### Live-Dead Cell Staining

BM-MSC spheroids were washed with PBS (−) and left to stand in a glass base dish (Iwaki, Haibara, Shizuoka, Japan). Spheroids were stained with a LIVE/DEAD Viability/Cytotoxicity kit (Invitrogen, Carlsbad, CA, United States) for 30 min at room temperature. Stained spheroids were observed using an LSM 780 confocal microscope (Carl Zeiss AG, Oberkochen, Germany).

### B -Galactosidase Staining (Cell Senescence)

β-Galactosidase (SA-β-GAL) activity was detected using the senescence detection kit (BioVision, Milpitas, CA, United States). Cells were fixed with a fixable solution (senescence detection kit, Bio Vision Milpitas) for 15 min at room temperature (approx. 25°C). Fixed cells were then washed with PBS (−) and treated with staining solution, staining supplements, and X-gal solution (final concentration: 1 mg/mL; BioVision, Milpitas, CA, United States), and incubated overnight at 37°C.

### Reverse transcription polymerase chain reaction (RT-PCR) Experiments

Total RNA was extracted from cells using the TRIzol reagent (Thermo Fisher Scientific, Waltham, MA, United States) and a RNeasy Mini kit (Qiagen, Hilden, Germany). Total RNA after 21 days of induction was normalized for all RT-PCR samples. The samples were treated with DNase I (Invitrogen) to remove any genomic DNA contamination. A reverse transcription reaction was conducted with random primers (Promega, Madison, WI, United States) and total RNA, according to the manufacturer’s instructions. Next, complementary DNA (cDNA) was synthesized from 1 μg of total RNA using a Reverse Transcription System (Promega). The cDNA target was amplified via PCR using Taq DNA polymerase (Promega), following the manufacturer’s recommendations (Niibe et al., 2011a). The primer pairs used for the RT-PCR experiments are described in Table 1. PCR products were subjected to 1% agarose gel electrophoresis with ethidium bromide staining and visualized under illumination with ultraviolet light (Dolphin-view 2, WEALTEC, Meadowvale Way Sparks, NV, United States).

**TABLE 1:** Primers used for RT-PCR.

	**Gene**	**Primer sequence (5′-3′)**	**Product size**	**Accession number**
Mouse	*Snail-F*	CTTGTGTCTGCACGACCTGT	167	NM_011427.3
	*Snail-R*	CTTCACATCCGAGTGGGTTT		
	*Twist-F*	GGAGGATGGAGGGGGCCTGG	225	NM_011658.2
	*Twist-R*	TGTGCCCCACGCCCTGATTC		
	*PDGFR*α*-F*	TACATCATCCCCCTGCCAGA	270	NM_011058.3
	*PDGFR*α*-R*	AAGGTTATCCCGAGGAGGCT		
	*Col1a2-F*	CCTGACGCATGGCCAAGAAGA	145	NM_007742.4
	*Col1a2-R*	GCATTGCACGTCATCGCACA		
	*OPN-F*	CAGTGATTTGCTTTTGCCTGTTTG	356	NM_001204203.1
	*OPN-R*	GGTCTCATCAGACTCATCCGAATG		
	*OCN-F*	GACCATCTTTCTGCTCACTCTG	276	NM_007541.3
	*OCN-R*	GTGATACCATAGATGCGTTTGTAG		
	*Adipsin-F*	ACTCCCTGTCCGCCCCTGAACC	433	NM_001329541.1
	*Adipsin-R*	CGAGAGCCCCACGTAACCACACCT		
	*PPAR*γ*-F*	TTCTGACAGGACTGTGTGACAG	355	NM_001127330.2
	*PPAR*γ*-R*	ATAAGGTGGAGATGCAGGTTC		
	*ACAN-F*	AAGTTCCAGGGTCACTGTTAC	269	NM_007424.2
	*ACAN-R*	TCCTCTCCGGTGGCAAAGAAG		
	*Sox9-F*	CCTTCAACCTTCCTCACTACAGC	131	NM_011448.4
	*Sox9-R*	GGTGGAGTAGAGCCCTGAGC		
	*Col2a1-F*	CCTCCGTCTACTGTCCACTGA	121	NM_001113515.2
	*Col2a1-R*	ATTGGAGCCCTGGATGAGCA		
	β*-actin-F*	CTGTCCCTGTATGCCTCTG	218	NM_007393.5
	β*-actin-R*	ATGTCACGCACGATTTCC		
	*GAPDH-F*	CACCATGGAGAAGGCCGGGG	418	NM_001289726.1
	*GAPDH-R*	GACGGACACATTGGGGGTAG		
Human	*Nestin-F*	CAGCGTTGGAACAGAGGTTGG	389	NM_006617.1
	*Nestin-R*	TGGCACAGGTGTCTCAAGGGTAG		
	*Slug-F*	AGATGCATATTCGGACCCAC	258	NM_003068.4
	*Slug-R*	CCTCATGTTTGTGCAGGAGA		
	*Vimentin-F*	TGTCCAAATCGATGTGGATGTTTC	117	NM_003380.4
	*Vimentin-R*	TTGTACCATTCTTCTGCCTCCTG		
	*Oct3/4-F*	GACAGGGGGAGGGGAGGAGCTAGG	144	NM_001285987.1
	*Oct3/4-R*	CTTCCCTCCAACCAGTTGCCCCAAAC		
	*Ki67-F*	TGACCCTGATGAGAAAGCTCAA	141	NM_002417.5
	*Ki67-R*	CCCTGAGCAACACTGTCTTTT		
	*B -actin-F*	CACCAACTGGGACGACAT	189	NM_001101.5
	*B -actin-R*	ACAGCCTGGATAGCAACG		
	*GAPDH-F*	GTCAAGGCCGAGAATGGGAA	613	NM_001256799.3
	*GAPDH-R*	GCTTCACCACCTTCTTGATG		
Human (Real-time RT-PCR)	*Sox2-F*	TGGTCCTGCATCATGCTGTAG	71	NM_003106.4
	*Sox2-R*	AACCAGCGCATGGACAGTTAC		
	*GAPDH-F*	GAAGGTGAAGGTCGGAGTCA	172	NM_001357943.2
	*GAPDH-R*	GAAGATGGTGATGGGATTTC		

### Statistical Analysis

All experimental data are presented as the mean ± standard derivation (SD). Statistical analysis was performed using one-way analysis of variance, followed by Tukey’s multiple-comparison test to assess differences among multiple experimental groups. Statistical significance was set at *P* < 0.05.

## Results

### Multipotency Loss of mBM-MSCs in Adherent Culture

**FIGURE 1:**
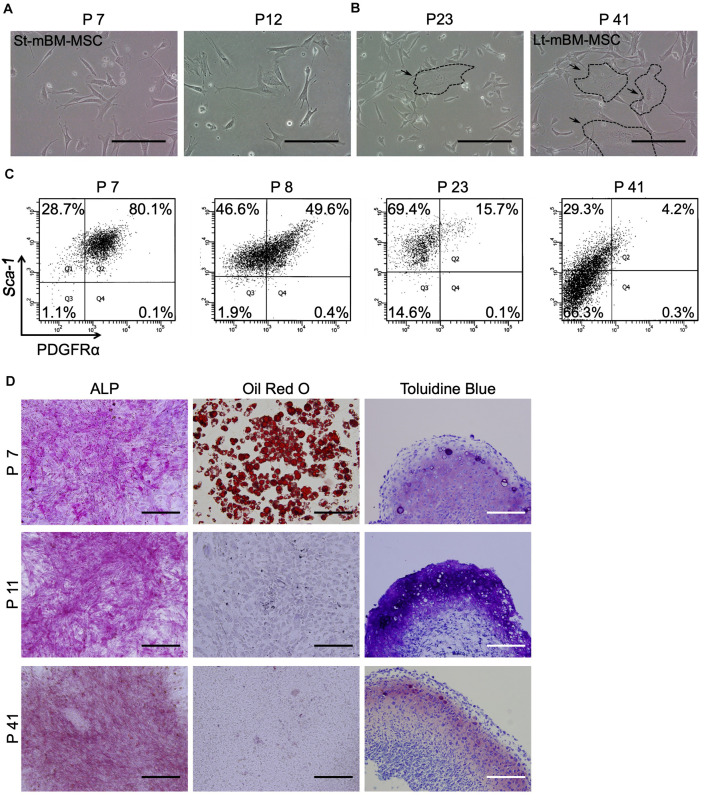
Phenotype of mouse MSCs in adherent culture. **(A)** Phase contrast image of mouse MSCs at P7 and P12 (St-mBM-MSCs) under adherent culture conditions. **(B)** Phase contrast images of mouse MSCs at P23 and P41 (Lt-mBM-MSCs) under adherent culture conditions. Broken line: flattened cell. **(C)** FACS profiles of mouse MSCs at P7, P8, P23, and P41. Horizontal axis: PDGFRα expression; vertical axis: Sca-1 expression. **(D)** Differentiation potential of mouse MSCs after P7, P11, and P41. Osteogenesis is indicated by ALP staining. Adipogenesis is indicated by neutral lipid vacuoles, which were stained with Oil red O. Chondrogenesis is indicated by toluidine blue staining. Scale bars: 200 μm.

We first characterized mBM-MSCs, grown as adherent cultures, in terms of their cell-surface expression of PDGFRα, and Sca-1 (markers of highly enriched mBM-MSCs), as well as their ability to differentiate toward a mesenchymal lineage (Morikawa et al., 2009a). mBM-MSCs that underwent a low number (7 to 12) of passages, i.e., short-term (St)-cultured mBM-MSCs, exhibited an elongated and spindle-shaped morphology (Figure 1A). In contrast, MSCs that underwent a high number (23 to 41) of passages, i.e., long-term (Lt)-cultured MSCs, became wider and lost their spindle shape (Figure 1B). Flow cytometric analysis showed that 80.1% of the mBM-MSC population at P7 consisted of highly enriched mBM-MSCs (PDGFRα^+^/Sca-1^+^ cells). However, this population gradually decreased to 49.6% at P8, to 15.7% at P23, and to 4.2% at P41 (Figure 1C). The mBM-MSCs at P7 showed capacity for tri-lineage differentiation into osteoblasts, adipocytes, and chondrocytes, as indicated by positive staining with ALP, Oil red O, and toluidine blue, respectively (Figure 1D, upper panels). However, mBM-MSCs at P11 and P41 acquired a bi-lineage capacity by losing their potential for adipogenic-differentiation (Figure 1D, middle and lower panels; and Supplementary Figure 1A). These results indicate the fragility of the tri-lineage differentiation ability of mBM-MSCs under adherent culture conditions following repeated passages in association with a decrease in PDGFRα^+^/Sca-1^+^ cells.

### The Shaking-Culture Method Generated Spheroids From mBM-MSCs

We then attempted to expand and maintain MSC spheroids using a low-attachment culture plate that was previously used in “mesensphere” studies (Baraniak and McDevitt, 2012). P25 MSCs were prepared to obtain mesenspheres with low-attachment culture dishes (Elplasia RB500 400 NA plate, Kuraray). P25 mBM-MSCs were divided into 1.5 × 10^6^ cells/mL and 2.5 × 10^6^ cells/mL. At day 0, mBM-MSCs were seeded into 500 × 400-μm micro spaces (Supplementary Figure 2A). After 7 days, seeded mBM-MSCs at all conditions developed MSC spheroids (Supplementary Figure 2A). However, the spheroids moved to another microspace and aggregated with others, during the changing of medium (Supplementary Figure 2A black arrow). Therefore, cells grown in the low-attachment culture dish did not maintain their rounded shape in the individual microspaces, and it was not possible to regularly change the medium. After 7 days of culture in a low-attachment culture dish, spheroids were then re-seeded into an adherent plastic culture dish (Supplementary Figure 2B). At day 0, after re-seeding on the adherent culture dish, the spheroids of all conditions maintained their round shape morphology. However, 3 days after re-seeding, the spheroids lost their 3D round shape. At day 7, all cells returned to the spindle-shaped morphology (Supplementary Figure 2B). Further, we determined their differentiation potential. These cells also lost their adipogenic potential (21 days induction), similarly to the mBM-MSCs cultured using the adherent culture technique, even 3 days after re-seeding MSC spheroids on an adherent culture dish (Supplementary Figure 2C). In addition, mouse P41 mBM-MSCs were unable to recover their adipogenic-differentiation potential (data not shown). Moreover, enriched hBM-MSCs were unable to acquire floating spheroids in the low-attachment culture dish, even when P7 hBM-MSCs with normal tri-lineage-differentiation potential were used (Supplementary Figure 2D). Meanwhile, hBM-MSCs were attached and combined on the low-attachment culture dish (Supplementary Figure 2D, white arrow). These results demonstrate that the differentiation potential of BM-MSCs was not maintained using a low-attachment culture dish. Hence, we attempted to establish a novel culture method to obtain stable MSC spheroids that maintain their differentiation potential.

**FIGURE 2:**
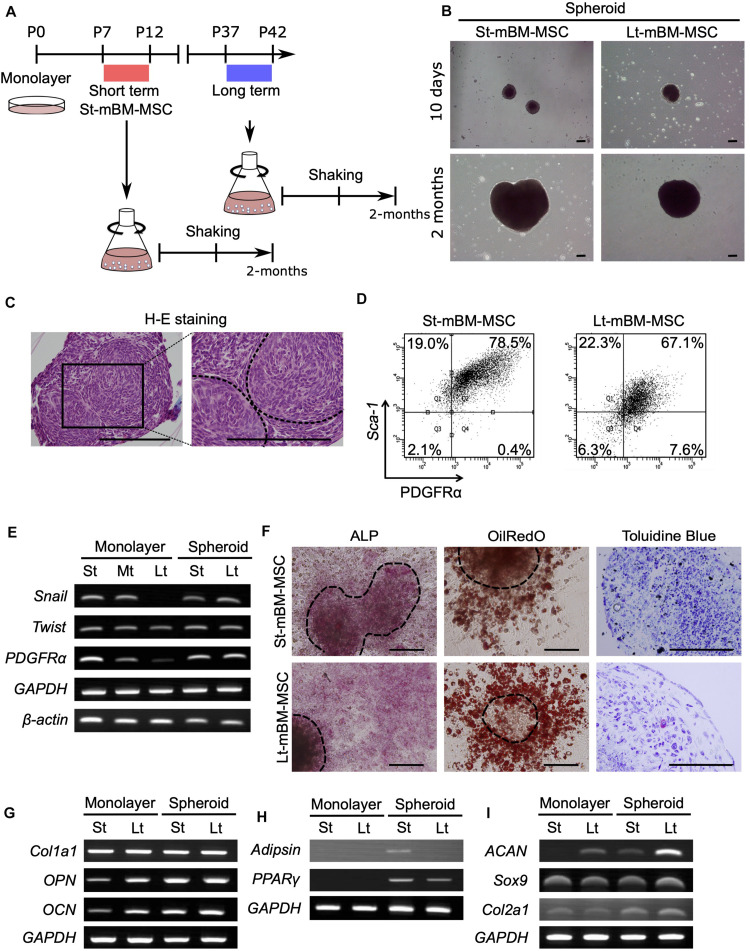
Phenotype of mouse MSC spheroids in the shaking-culture system. **(A)** Schematic of the mouse MSC shaking-culture system. St-mBM-MSCs consisted of mouse BM-MSCs grown from P7 to P12, whereas Lt-mBM-MSC consisted of mouse BM-MSCs grown from P37 to P42. **(B)** Mouse MSC spheroids after 10 days or 2 months of shaking. **(C)** H-E staining of St-mBM-MSC spheroids. Broken line indicates small spheroids. **(D)** FACS profiles of St-mBM-MSC spheroids and Lt-mBM-MSC spheroids following 2 months of growth in shaking-culture. Horizontal axis: PDGFRα expression; vertical axis: Sca-1 expression. **(E)** RT-PCR analysis of MSC spheroids compared with adherent monolayered MSCs. The St-MSCs used were P7, the middle (Md) MSCs were P25, and the Lt-MSCs were P37. MSC marker genes: *PDGFR*α and *Sox9*. Neural crest stem cell-marker genes: *Snail* and *Twist*. **(F)** Differentiation potential of MSC spheroids. Osteogenesis is indicated by ALP staining. Adipogenesis is indicated by neutral lipid vacuoles, which were stained with Oil red O. Chondrogenesis is indicated by toluidine blue staining. Upper panel: St-mBM-MSCs spheroids; lower panel: Lt-mBM-MSCs spheroids. **(G)** RT-PCR analysis of the osteogenic-differentiation assay in terms of the osteogenic marker genes *Col1a1*, *OPN*, and *OCN*. **(H)** RT-PCR analysis of the adipogenic-differentiation assay in terms of the adipogenic marker genes *Adipsin* and *PPAR*γ. **(I)** RT-PCR analysis of chondrogenic-differentiation assay in terms of the chondrogenic marker genes *ACAN*, *Sox9*, and *Col2a1*. Scale bars: 200 μm.

Shaking devices are widely used to culture microorganisms and animal cells for recombinant protein expression and cell mass production (Liu and Hong, 2001). However, no reports have described the culturing of MSCs under shaking-culture conditions to fabricate spheroids. We conducted shaking cultures of both St-mBM-MSCs (P7–12) and Lt-mBM-MSCs (P37–42) using 1 × 10^7^ cells in each case, and 125-mL flasks (Figure 2A).

Small visible spheroids appeared after 10 days of growth in shaking-culture (Figure 2B: upper panels). The numbers of spheroids present after 10 days of shaking-culture in St-mBM-MSCs and Lt-mBM-MSCs were 34.3 ± 10.5 (mean ± SD; *n* = 3) and 29.6 ± 5.5 (mean ± SD; *n* = 3), respectively. The spherical morphology of these cells persisted after culture for 2 months (Figure 2B; lower panels). H&E-stained spheroid sections following 1 month of shaking-culture showed that they were composed of bordered cell aggregates surrounded by a layer of homogeneous compact cells (Figure 2C). These results implied that, under shaking-culture conditions, small spheroids bind to each other via collision to form larger spheroids. Following 2 months of shaking-culture, the Feret’s diameters of St-mBM-MSCs and Lt-mBM-MSCs spheroids were 908.8 ± 258.8 μm (mean ± SD, *n* = 25) and 779.25 ± 195.1 μm (mean ± SD, *n* = 10), respectively.

Furthermore, 78.5% of spheroids from St-mBM-MSCs grown for 2 months in shaking-culture consisted of highly enriched mBM-MSCs (PDGFRα^+^/Sca-1^+^ cells). Notably, 67.1% of these spheroids was still maintained even after Lt-mBM-MSCs were cultured for 2 months under shaking conditions (Figure 2D). RT-PCR analyses showed that the expression levels of an MSC marker (*PDGFR*α) and two NCSC markers (*Snail* and *Twist*) clearly decreased following repeated passage of mBM-MSCs in adherent culture (Figure 2E), suggesting that the Lt-mBM-MSCs had lost the intrinsic gene expression profile of highly enriched mBM-MSCs, due to monolayer expansion. When St-mBM-MSCs were grown under shaking-culture conditions for 2 months, the expression of these genes (indicative of highly enriched mBM-MSCs) was maintained in the spheres. In addition, the expression levels of *PDGFR*α and *Snail* were upregulated in shaking-culture when Lt-mBM-MSCs were used for sphere fabrication (Figure 2E).

Both St-mBM-MSC- and Lt-mBM-MSC-derived spheroids grown for 2 months under shaking-culture conditions showed tri-lineage (osteogenic, adipogenic, and chondrogenic)-differentiation capacities compared with undifferentiated MSC-derived spheroids (Figure 2F and Supplementary Figure 1B). ALP staining and Oil red O staining following a 21-day induction indicated that cells comprising, and growing from, the spheroids robustly differentiated into osteoblasts and adipocytes, respectively. Notably, Lt-mBM-MSCs in spheroids restored the adipogenic-differentiation capacity of Lt-mBM-MSCs that had been previously lost due to repeated passages under adherent culture conditions (see Oil red O staining results in Figure 1D). The tri-lineage gene expression profiles of Lt-mBM-MSC-derived spheres were confirmed via RT-PCR. Robust expression of osteogenic marker genes, such as collage type Iα1 (*Col1a1*), osteopontin (*OPN*), and osteocalcin (*OCN*), was confirmed in both St-mBM-MSC- and Lt-mBM-MSC-derived spheroids following a 21-day induction (Figure 2G and Supplementary Figure 3). Following a 21-day adipogenic induction, the expression of adipogenic marker genes, such as complement factor D (*Adipsin*) and peroxisome proliferator-activated receptor γ (*PPAR*γ), was not detected in St-mBM-MSCs and Lt-mBM-MSCs grown under adherent culture conditions. In contrast, adipsin and *PPAR*γ expression was clearly confirmed in St-mBM-MSC- and Lt-mBM-MSC-derived spheroids (Figure 2H and Supplementary Figure 3). Regarding their chondrogenic-differentiation capacity, mBM-MSC spheroids fabricated from Lt-mBM-MSCs, in particular, expressed the chondrogenic marker genes, aggrecan (*ACAN*), *Sox9*, and collagen type-IIα1 (*Col2a1*) at higher levels than cells grown under adherent culture conditions (Figure 2I and Supplementary Figure 3). These results indicate that the shaking-culture conditions maintained the stemness of mBM-MSCs in spheroidal form and even restored the adipogenic-differentiation capacity of Lt-mBM-MSCs.

### Fabrication of hBM-MSC Spheroids Using the Shaking-Culture Method

**FIGURE 3:**
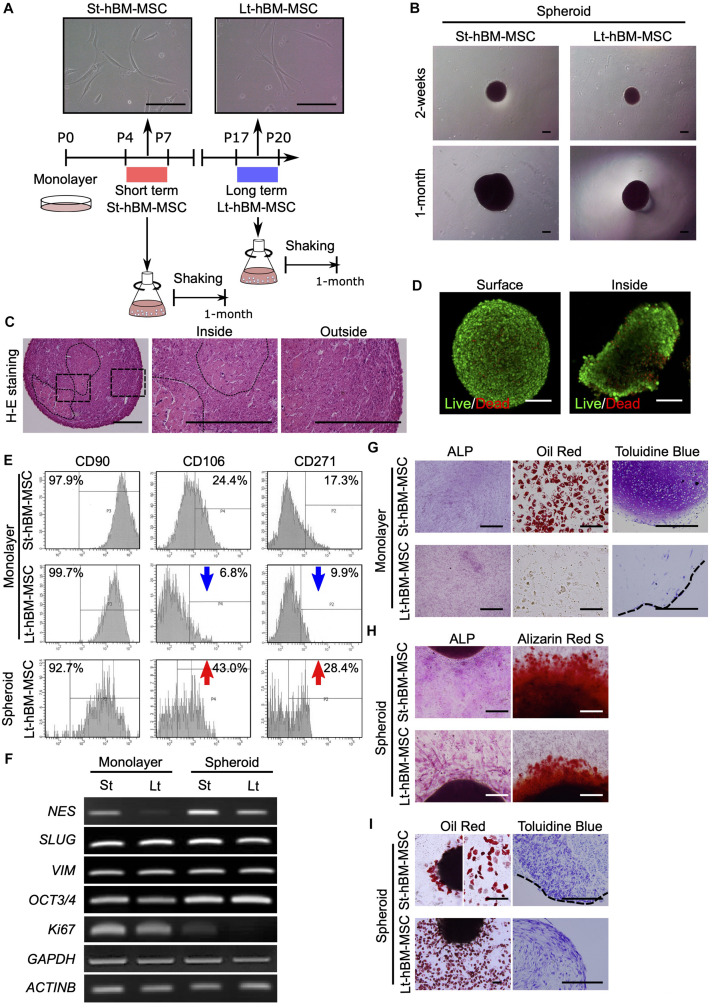
Phenotype of human MSC spheroids in the shaking-culture system. **(A)** Schematic of the human MSC shaking-culture system. Phase contrast images of St-hBM-MSCs (P4–P7) and Lt-hBM-MSCs (P17–P20) on adherent plastic dishes. **(B)** St-hBM-MSCs and Lt-hBM-MSCs after 2 weeks and 1 month of shaking. **(C)** H-E staining of St-hBM-MSC-derived spheroids. The magnified images indicate the inside and outside layers of the spheroids. Broken line indicates small spheroids. **(D)** Live/dead staining of St-hBM-MSC-derived spheroids. **(E)** FACS profiles of human MSCs and MSC spheroids. Left panel: CD90 expression; middle panel: CD106 expression; right panel: CD271 expression. **(F)** RT-PCR analysis of MSC spheroids compared with adherent MSCs. MSC and neural crest stem cell-marker genes: *NES*, *SLUG*, and *VIM*. Cell proliferation marker: *Ki67*. Stem cell-marker gene: *OCT3/4*. **(G)** Differentiation potential of monolayered human MSCs. Broken line: surface of chondrogenic pellet. **(H)** Differentiation potential of MSC spheroids. Osteogenesis is indicated by ALP staining and Alizarin red S staining. **(I)** Adipogenesis is indicated by neutral lipid vacuoles, which were stained with Oil red O. Chondrogenesis is indicated by toluidine blue staining. Broken line: surface of chondrogenic pellet. Scale bars: 200 μm.

Next, we attempted to obtain spheroids from highly enriched hBM-MSCs (CD271^+^/CD90^+^ cells). To this end, CD271^+^/CD90^+^ hBM-MSCs were isolated using fluorescence activated cell sorting (FACS) and expanded under adherent culture conditions until P20. These hBM-MSCs maintained a spindle-shaped morphology through P20 (Figure 3A). However, some populations of cells in Lt-hBM-MSC progressively grew wider, and cell senescence occurred. Cells in 11.92% (*n* = 4) of St-hBM-MSCs were positive for SA-β-gal staining, and Lt-hBM-MSCs were increasingly positive for 33.98% (*n* = 4, Supplementary Figures 4A,B). Besides, hBM-MSCs showed a markedly reduced proliferation speed and CD106 (<10%) and CD271 (<10%) expression levels after P17. When 1 × 10^6^ hBM-MSCs at low (P4–7: St-hBM-MSCs) or high (P17–20: Lt-hBM-MSC) passage numbers were grown in shaking-culture in 125-mL flasks, they produced spheroids within 14 days (Figure 3B). After a month of growth under shaking-culture conditions, St-hBM-MSCs and Lt-hBM-MSCs produced 14.3 ± 2.0 (mean ± SD; *n* = 3) and 21.3 ± 7.0 (mean ± SD; *n* = 3) spheroids, respectively. Feret’s diameters of the St-hBM-MSC and Lt-hBM-MSC spheroids were 699.0 ± 205.4 μm (mean ± SD; *n* = 5) and 534.2 ± 269.1 μm (mean ± SD; *n* = 6), respectively. After 1 month in culture, no statistically significant difference was found between the spheroid numbers and Feret’s diameters when starting with 1 × 10^6^ or 1 × 10^7^ cells (Table 2 and Supplementary Figure 5). After 2 months of shaking-culture, the sizes of both the St-hBM-MSC and Lt-hBM-MSC spheroids did not increase significantly (Table 2 and Supplementary Figure 5). However, it is important to reduce the culture period for the clinical application of human MSC spheroids, therefore, a 1 month shaking was chosen in the following study.

**TABLE 2:** Effects of shaking-culture on the numbers and sizes of hBM-MSC spheroids.

		**Sphere number**		**Feret’s diameter (μ m)**	
		**1 month**	**2 months**	**1 month**	**2 months**
St-hBM-MSCs	1 × 10^6^	14.3 ± 2.0 (*n* = 3)	10.7 ± 2.0 (*n* = 3)	699.0 ± 205.4 (*n* = 5)	756.7 ± 185.5 (*n* = 6)
	1 × 10^7^	15.0 ± 1.0 (*n* = 3)	11.7 ± 2.5 (*n* = 3)	782.4 ± 342.9 (*n* = 17)	833.3 ± 184.2 (*n* = 6)
Lt-hBM-MSCs	1 × 10^6^	21.3 ± 7.0 (*n* = 3)	17.0 ± 4.6 (*n* = 3)	534.2 ± 269.2 (*n* = 6)	733.8 ± 193.2 (*n* = 5)
	1 × 10^7^	23.3 ± 8.5 (*n* = 3)	17.3 ± 4.5 (*n* = 3)	759.9 ± 369.5 (*n* = 9)	1158.3 ± 225.1 (*n* = 6)

*St-hBM-MSCs were cultured between P4 and P7. Lt-hBM-MSCs were cultured between P17 and P20.*

H&E staining of St-hBM-MSC-derived spheroid sections after 1 month of shaking-culture indicated that these spheroids had formed cell aggregates by binding to each other (Figure 3C). The inner regions of the spheroids contained areas of abundant extracellular matrix (ECM), surrounded by homogeneous, compact cells. Live/dead staining after 4 weeks of shaking-culture showed that most cells on the spheroid surface and outer layer were alive (stained in green). Although fewer cells were present in the central region of the spheroids (due to an abundant ECM), most of the cells were alive (Figure 3D). Moreover, the SA-β-gal staining of migrated cells from Lt-hBM-MSC spheroids attached after 3 days on the adherent culture dish, decreased to 11.46% (*n* = 4; Supplementary Figure 4B).

Flow cytometry indicated that, under all culture conditions, a high percentage of hBM-MSCs (>90%) showed high expression levels of CD90, a representative MSC marker (Figure 3E). Next, we analyzed the expression levels of the MSC markers, CD106 and CD271, which are preferentially expressed in highly enriched hBM-MSCs (Mabuchi et al., 2013). CD106 expression in monolayer hBM-MSCs decreased markedly from 24.4% in St-hBM-MSCs to 6.8% in Lt-hBM-MSCs, following repeated passages. CD106 expression in Lt-hBM-MSCs clearly increased up to 43% under shaking-culture conditions. Under monolayer culture conditions, St-hBM-MSCs exhibited 17.3% CD271-positive cells, which decreased to 9.9% in Lt-hBM-MSCs. CD271 expression in Lt-hBM-MSCs increased markedly up to 28.4% under shaking-culture conditions (Figure 3E).

Our RT-PCR results showed that although St-hBM-MSCs maintained their expression levels of MSC markers, NESTIN (*NES*) (Xie et al., 2015), SNAI2 *(SLUG)* (Torreggiani et al., 2012), and VIMENTIN *(VIM)* (Zhang et al., 2018) under both monolayer and shaking-culture conditions (Figure 3F). The two housekeeping genes were investigated to define their gene expression patterns. *GAPDH* and β-ACTIN (*ACTNB)* expression did not significantly differ between monolayer MSCs and spheroids (Figure 3F). The monolayer Lt-hBM-MSCs markedly lost *NES* expression. By contrast, *NES* expression was restored in Lt-hBM-MSC-spheroids after 1 month of shaking-culture. Interestingly, expression of the primitive stem cell-marker, *OCT3/4*, clearly increased in monolayer hBM-MSCs under shaking-culture conditions. In addition, the pluripotent marker *SOX2* (Zhang and Cui, 2014) expression was decreased in monolayer LT-hBM-MSCs, and increased in spheroids (Supplementary Figure 6A). Meanwhile, *Ki67* expression (Bruno and Darzynkiewicz, 1992), known as a marker of cell proliferation was decreased in spheroids (Figure 3F).

Next, the capacity of hBM-MSCs for differentiation into a mesenchymal cell lineage was investigated. St-hBM-MSC and Lt-hBM-MSC spheroids were positive for ALP and Alizarin red S staining following osteogenic induction after 1 month shaking (Figure 3H and Supplementary Figure 6C). Monolayer Lt-hBM-MSCs did not form lipid droplets after adipogenic induction (Figure 3G and Supplementary Figure 6B) and did not form 3D chondrogenic pellets after repeated induction. Therefore, we used adherent cultured cells for staining negative controls (Supplementary Figure 6B). The 3D pellet from monolayer Lt-hBM-MSCs stained negative with toluidine blue after chondrogenic-induction. After 1 month shaking-culture, Lt-hBM-MSCs showed a capacity for adipogenic and chondrogenic-differentiation, based on Oil red and toluidine blue staining, respectively (Figure 3I and Supplementary Figure 6C), suggesting that the shaking-culture had restored the capacity for tri-lineage differentiation. A previous study showed that enriched mBM-MSC (Morikawa et al., 2009b; Niibe et al., 2011b) and hBM-MSCs (Niibe et al., 2017) can differentiate into neuron-like cells. We attempted to examine spheroids’ neuronal differentiation potential. Monolayer St-hBM-MSCs showed βIII tubulin^+^ neuron-like cells after neurogenic induction, and neurogenic differentiation capacity seemed to be maintained even after 1 month under shaking-culture conditions (Supplementary Figure 7). From these results, our shaking-culture method seems to preserve the undifferentiated state of hBM-MSCs.

### “Undifferentiated MSC-Pool” Characteristics Are Properties of the hBM-MSC Spheroids

We successfully generated “mesenspheres” using a low-attachment culture dish (Elplasia RB500 400 NA plate, Kuraray) with P25 mBM-MSCs (Supplementary Figure 1) and P41 mBM-MSCs (data not shown). However, the mesenspheres re-seeded onto an adherent plastic culture dish lost their 3D round shape. At day 7, all cells returned to the spindle shape morphology (Supplementary Figure 2B). In addition, enriched hBM-MSCs were unable to acquire floating spheroids in the low-attachment culture dish, even when P7 hBM-MSCs with standard tri-lineage differentiation potential were used (Supplementary Figure 2D). hBM-MSCs were attached and combined on the low-attachment culture dish (Supplementary Figure 2D white arrow). Meanwhile, re-attached hBM-MSC spheroids produced by the shaking-culture method provided undifferentiated cells and maintained their round shape after osteogenic and adipogenic-differentiation (Figure 3H). Moreover, CD106 expression, a vascular cell-adhesion protein (Figure 3E), increased in hBM-MSC spheroids. Hence, hBM-MSC spheroids seemingly provide a robust connective ability that preserves the undifferentiated hBM-MSCs from attached spheroids.

**FIGURE 4:**
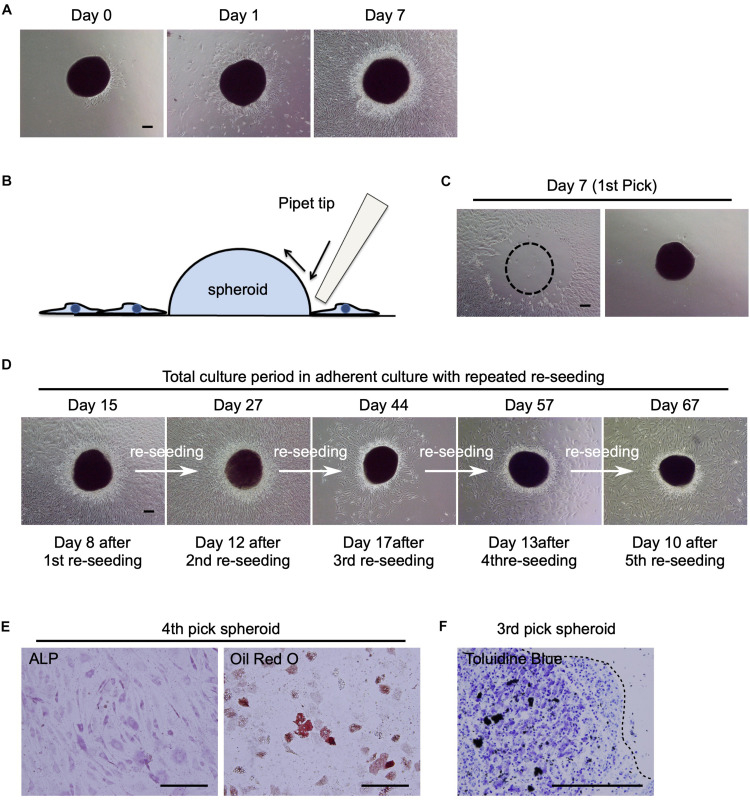
Potential of human MSC spheroids as a stem cell pool. **(A)** Phase contrast images of St-hBM-MSC-derived spheroids obtained by shaking at day 0, 1, and 7 after re-seeding into an adherent culture dish. **(B)** Schematic representation of the method for picking up MSC spheroids. **(C)** Phase contrast images of MSC spheroids after the 1st pick-up and re-seeding to a different culture plate. Left panel: culture plate after picking up of spheroids; right panel: culture plate after re-seeding MSC spheroids. Broken line indicates position of spheroid after harvest. **(D)** Phase contrast images of re-seeded MSC spheroids until the 5th re-seeding. **(E)** Differentiation potential of human MSC spheroids after the 4th pick-up. Osteogenesis is indicated by ALP staining. Adipogenesis is indicated by neutral lipid vacuoles, which were stained with Oil red O. **(F)** Differentiation potential of human MSC spheroids after the 3rd pick-up. Chondrogenesis is indicated by toluidine blue staining. Broken line: surface of chondrogenic pellet. Scale bars: 200 μm.

To continuously harvest MSCs from the spheroids, St-hBM-MSC-spheroids (obtained after 1 month of growth in shaking-culture) were re-seeded in the cell culture dish in a static condition. The spheroids re-attached to the cell culture dish within a day (Figure 4A; Day 0), and many cells migrated from the spheroids on day 1 (Figure 4A; Day 1). Interestingly, the attached spheroids continued to produce migratory cells even on day 7 (Figure 4A: Day 7), while maintaining their size and rounded shape.

Next, we investigated whether the attached spheroids could sustain the ability to produce migratory hBM-MSCs (re-seeding capacity) by transferring them to new cell culture plates. The spheroids were mechanically harvested using pipette tips (Figure 4B) and seeded in new cell culture dishes (Figure 4C). The spheroids easily re-attached to new culture dishes within a day and produced migratory cells after 8 days (Figure 4D; Day 15). Interestingly, when the spheroids were repeatedly harvested and re-seeded four more times, they continued to provide migratory cells in abundance (Figure 4D; Day 27–67). This re-seeding capacity was also observed in mBM-MSC spheroids (data not shown).

To confirm the multilineage differentiation capacity of the migratory cells, migrated cells derived from re-seeded hBM-MSC-spheroids were cultured in osteogenic, adipogenic, or chondrogenic-induction medium. After a 21-day induction period, migratory cells showed ALP^+^ osteoblasts, Oil red O^+^ adipocytes, and toluidine blue^+^ chondrocytes (Figures 4E,F), suggesting the multipotent capacity of these cells as MSCs. These results suggest that hBM-MSC spheroids, which were obtained by the shaking-culture method, showed potential as a high-quality “MSC-pool.”

## Discussion

Conventionally, MSCs expand into adherent monolayers, however, eventually lose their ability to proliferate and differentiate into mesenchymal lineages (Bonab et al., 2006; Stolzing et al., 2006; Wagner et al., 2008; Bork et al., 2010). This phenotypic alteration also observed in the case of “enriched hMSCs” (Figure 3). Our results indicate that the expression levels of specific MSC markers decreased in mouse (PDGFRα) and human-enriched MSCs (CD106), which lost their tri-lineage differentiation potential following several passages (Figures 1, 3). Therefore, the possibility of MSCs failing as a regenerative therapy remains, showing the necessity for a reliable number of cells following repeated passages when using conventional adherent culture systems. Here, we characterized St-MSCs and Lt-MSCs at different passage numbers, based on their proliferative ability, cell-surface markers, and differentiation potential, using both mBM-MSCs and hBM-MSCs.

To address the limitations associated with conventional adherent cultures and facilitate scaffold-free cell transplantation, a new culture system of MSCs using ultra-low-attachment culture dishes was recently developed to obtain MSC spheroids, which were referred to as “mesenspheres” (Mendez-Ferrer et al., 2010; Baraniak and McDevitt, 2012; Isern et al., 2013). MSC spheroids are considered as a promising tool for regenerative medicine (Jaukovic et al., 2020). The culture dishes in these studies had a predetermined microwell size at the bottom, which resulted in mesenspheres with a fixed size. Human mesenspheres differentiated robustly into mesenchymal lineages and enhanced the expansion of cord blood CD34^+^ cells (Isern et al., 2013). Unfortunately, herein, mouse mesenspheres obtained using low-attachment culture dishes with P25 and P41 MSCs did not recover their adipogenic-differentiation potential (Supplementary Figure 2). In addition, enriched hBM-MSCs did not acquire floating spheroids in the low-attachment culture dishes, even when P7 MSCs with normal tri-lineage differentiation potential were used. Furthermore, some mouse mesenspheres were transferred to other microwells during the medium changes and combined with each other. Thus, a potential limitation of the culture period with the floating culture system is the difficulty in maintaining a consistent size and shape over extended periods of time (Supplementary Figure 2). Further, the phenotypic details of spheroids following long-term culturing are not yet known. These study results, therefore, suggest that MSCs require a novel culture system to maintain their 3D round shape and need to evaluate their features and phenotype, such as cell viability, proliferation, or differentiation ability for use in regenerative therapies.

The shaking-culture technique has been widely used to expand microorganism cultures. Despite a previous report demonstrating that certain types of mechanical stress may enhance the stemness and differentiation potential of stem cells, no studies have investigated the possibility of culturing MSCs via the shaking-culture technique (Chen et al., 2016). In addition, certain types of mechanical stimulation induce pluripotency or enhance the differentiation potential of stem cells (Meier and Lam, 2016). Therefore, we hypothesized that MSCs are affected by shaking-culture conditions, by contacting each other, or by contacting the flask wall. Both mBM-MSCs and hBM-MSCs cultured in shaking flasks yielded MSC spheroids after 10 days. The resulting spheroids could maintain their round shape even after being continuously cultured for over 2 months. The spheroids were generated in an 85–95 rpm shaking condition with MSCs and spheroids found to be attached to the flask bottom at 85 rpm. In addition, the spheroids attached to the side wall when exposed to shaking over 95 rpm. We, therefore, successfully obtained spheroids 85–95 rpm shaking conditions.

Mesenspheres were reported to exhibit a similar proliferative capacity compared with that of conventional monolayers (Baraniak and McDevitt, 2012). However, under shaking-culture conditions, small spheroids bind to each other via collisions, forming larger spheroids, both with mouse and human BM-MSCs. Therefore, in our study, neither mBM-MSC spheroids nor hBM-MSC spheroids multiplied or effectively expanded MSCs after shaking. The hBM-MSC-spheroids decreased *Ki67* expression (Figure 3F). These results suggest that the shaking-culture system mimicked a state of quiescence or stopped the MSCs’ cell cycle. In addition, the spheroids stably retained their rounded shape and sunk to the flask bottom during the medium changes. Compared with the mesenspheres, our shaking system can provide a simple method for changing the medium.

Interestingly, FACS-sorted hBM-MSCs did not yield stable floating MSC spheroids after growth in low-attachment culture dishes (Supplementary Figure 2D). However, we successfully generated MSC spheroids using the shaking-culture method. hBM-MSC spheroids continuously yielded spindle-shaped monolayer MSCs and expanded markedly after re-seeding under adherent culture conditions and maintained their 3D round shape (Figure 4). This finding suggests that the highly adhesive nature of enriched hBM-MSCs may be associated with the expression of CD106, a cell-adhesion molecule. Generally, physical factors, such as the cell shape, external mechanical forces, ECM, and geometric structures regulate the lineage commitment of MSCs (Chen et al., 2016). In embryonic stem cells, cyclic mechanical strain enhances the expression of an essential regulator of stemness, such as Nanog expression (Horiuchi et al., 2012). Our hBM-MSC spheroids showed increased *OCT3/4* expression, a primitive stem cell-marker (Figure 3F), and maintained *SOX2* expression, a well-known pluripotency marker (Supplementary Figure 6A).

Our MSC spheroids maintained a rounded shape morphology after re-seeding in adherent culture dishes, and could be mechanically harvested and re-seeded repeatedly, and yielded fresh spindle-shaped MSC monolayers (Figure 4). In addition, these cells maintained their osteogenic, adipogenic, and chondrogenic-differentiation potentials.

We observed a slight increase in the size of mouse and human MSC spheroids; however, the increase was not significant. Meanwhile, the number of spheroids was found to be slightly decreased. These results imply that, under shaking-culture conditions, small spheroids bind to each other via collisions, to form larger spheroids in both mouse and human MSC spheroid cultures (Figures 2C, 3C). In addition, we attempted to count cell numbers and generate a growth curve. However, we were unable to dissociate spheroids either physically/mechanically or using enzymatic treatment. Therefore, we determined the number of spheroids and spheroid size, instead of generating a growth curve. MSC spheroids have a unique microenvironment compared with adherent MSCs, such as metabolic change or epigenetic regulation (Jaukovic et al., 2020). Other reports have shown that engineering MSC spheroids potentiated cell-cell and cell-ECM interaction (Lee et al., 2018). Hence, we postulate that the shaking-culture condition has the potential to alter the microenvironment of MSC spheroid to one suitable for maintaining the undifferentiated state. In addition, our previous study demonstrated that hypoxic conditions enhanced cell proliferation and adipogenic/osteogenic differentiation abilities of enriched mouse MSCs (Sato et al., 2016); therefore, in this study, a possible hypoxic condition in the MSC spheroids might affect the retention of the differentiation ability.

In this study, we attempted to produce both mBM-MSC and hBM-MSC spheroids with an undifferentiated state. We believed that enriched BM-BM-MSCs populations provide a pure MSC phenotype compared with the heterogeneous population supplied by whole bone marrow stromal cells. Our findings suggest that a shaking 3D culture maintained the multilineage potential of BM-MSCs and even restored the multipotency caused by monolayer expansion. Mouse MSC studies have the potential for implementation in basic research, such as a lineage-specific analysis using transgenic mice. Besides, human MSC spheroids have the potential to provide undifferentiated MSCs for clinical application. Human MSC spheroids also maintained their 3D shape for 2 months, and we confirmed their tri-lineage differentiation potential (data not shown). In the clinic, patients with severe conditions might be unable to wait 2 months to receive MSC transplantation therapies. For this reason, we tried to shorten the culture periods of human MSC spheroids for clinical application. Our novel spheroid-culture method shows potential as a promising strategy for use in regenerative medicine.

Taken together, these findings demonstrate the successful establishment of a 3D scaffold-free culture system, which enabled BM-MSCs to maintain an undifferentiated state and recover the *in vitro* multipotent differentiation potential of older BM-MSCs. Moreover, the results indicate that MSC spheroids can potentially provide fresh undifferentiated BM-MSCs, thereby forming an “undifferentiated MSC-pool” that could be used as a source of MSCs in regenerative medicine. However, further research is needed to determine the mechanism underlying the processes associated with recovering an undifferentiated state via shaking cultures.

## Data Availability Statement

The raw data supporting the conclusions of this article will be made available by the authors, without undue reservation.

## Ethics Statement

The animal study was reviewed and approved by Ethics Committee of Tohoku University in accordance with the Guide for the Care and Use of Laboratory Animals (approval identification numbers 2015DnA-022 and 2018DnA-002).

## Author Contributions

KN, YO-M, MZ, and YoM performed the experiments. KN, YO-M, and HE analyzed the results. YuM and HE provided scientific advice and materials and conceived the project and supervised the research. KN and HE wrote the manuscript. All authors contributed to the article and approved the submitted version.

## Conflict of Interest

The authors declare that the research was conducted in the absence of any commercial or financial relationships that could be construed as a potential conflict of interest.
