# Supplementary material for: A Shaking-Culture Method for Generating Bone Marrow Derived Mesenchymal Stromal/Stem Cell-Spheroids With Enhanced Multipotency in vitro
Source: Front Bioeng Biotechnol. 2020 Oct 20;8:590332. doi: 10.3389/fbioe.2020.590332 (PMC7641632; doi:10.3389/fbioe.2020.590332)
Supplement: Supplementary file 1 [file Data_Sheet_1.PDF]

Supplemental Information

Supplemental Figure 1

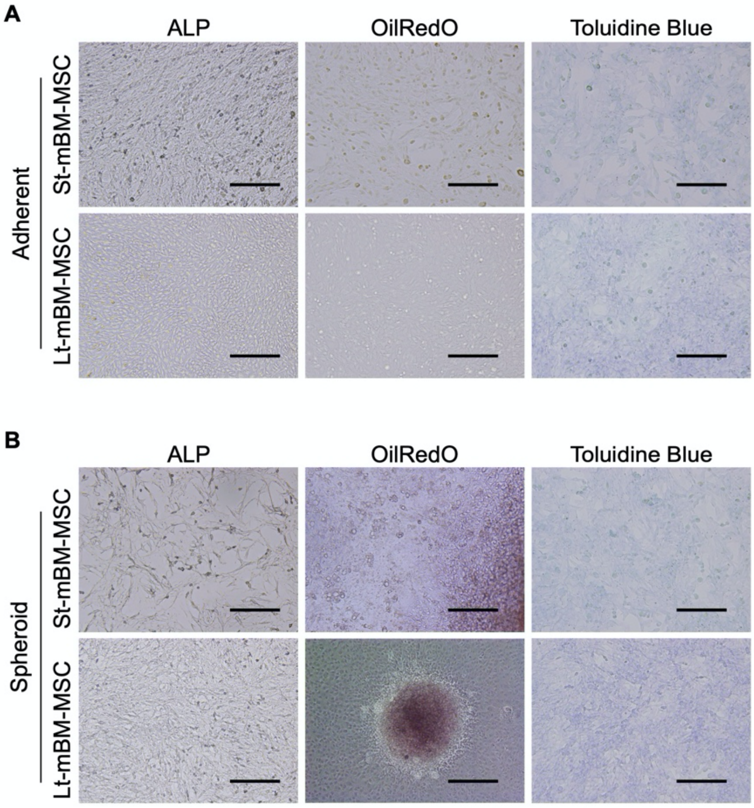

Supplemental Figure 1. Negative control staining for tri-lineage differentiation assay for mBM-MSCs. (**A**) Staining with adherent cultured St-mBM-MSC and Lt-mBM-MSC under maintenance condition. Osteogenesis is indicated by ALP staining. Adipogenesis is indicated by neutral lipid vacuoles, which were stained with Oil red O. Chondrogenesis is indicated by toluidine blue staining. (**B**) Staining with migrated cells from cultured St-mBM-MSC and Lt-mBM-MSC spheroids under maintenance condition. Osteogenesis is indicated by ALP staining. Adipogenesis is indicated by neutral lipid vacuoles, which were stained with Oil red O. Chondrogenesis is indicated by toluidine blue staining. Scale bars: 200 μm.

Supplemental Figure 2

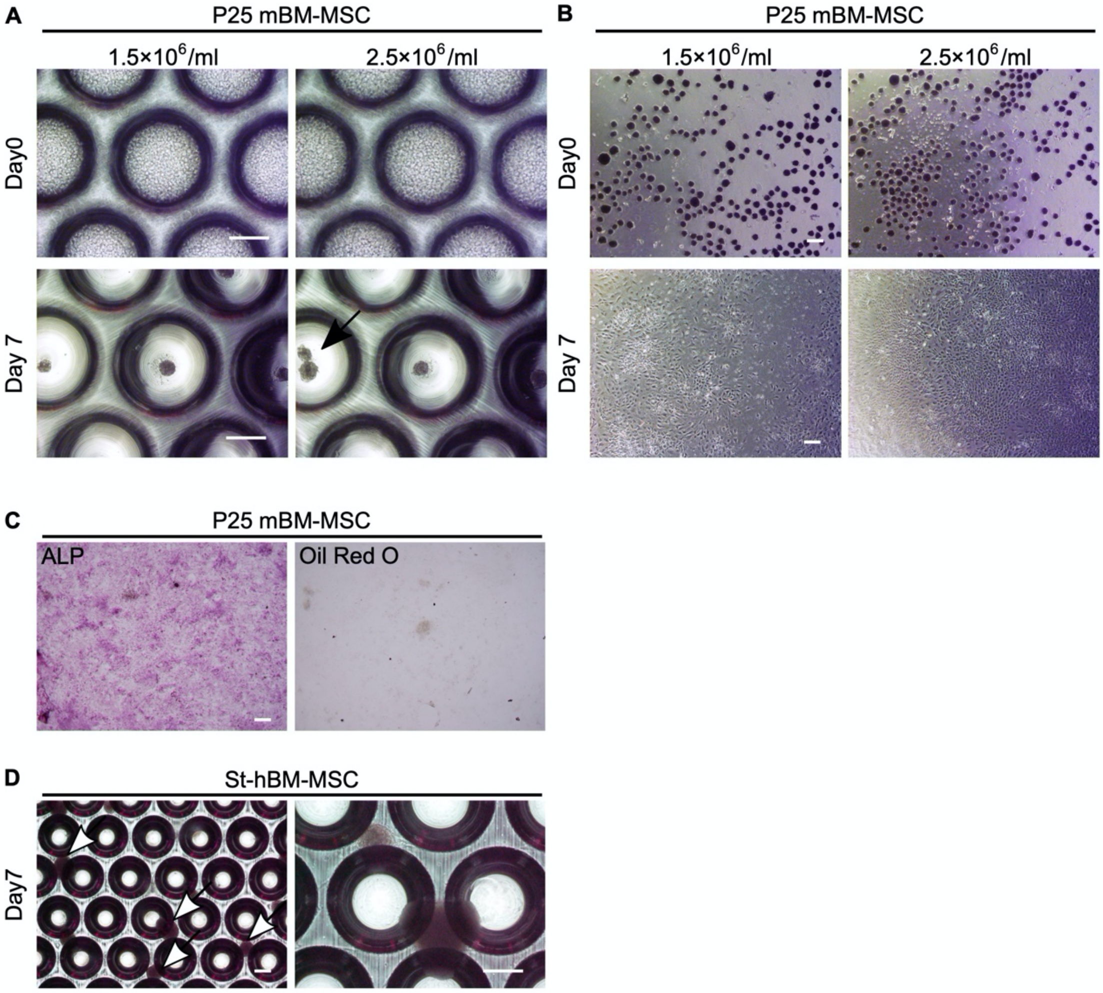

Supplemental Figure 2. Phenotype of mBM-MSC and hBM-MSC in a low-attachment dish. (**A**) Phase contrast of mBM-MSCs in a low-attachment culture dish (Elplasia #RB500 400 NA plate: Kuraray) at days 0 and 7 using P25 mBM-MSC. Black arrow indicates that spheroids moved to another microspace. (**B**) Phase contrast of re-seeded P25 mBM-MSCs spheroids in an adherent culture dish at days 0 and 7. (**C**) Differentiation potential of mBM-MSCs spheroids after culturing in an adherent culture dish. Osteogenesis was indicated by alkaline phosphatase (ALP) staining. Adipogenesis was indicated by neutral lipid vacuoles stained by Oil red O. (**D**) Phase contrast of hBM-

MSCs in a low-attachment culture dish (Elplasia #RB500 400 NA plate: Kuraray) at day 7, using P7 St-hBM-MSC. White arrow indicates attached and combined cells on a low-attachment culture dish. Scale bars: 200 μm.

Supplemental Figure 3

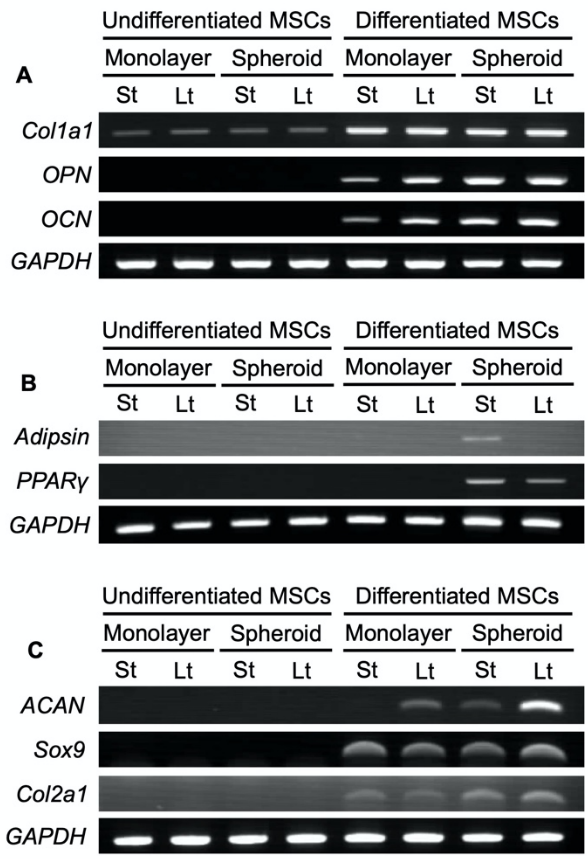

Supplemental Figure 3. RT-PCR analysis of differentiation assay for the undifferentiated MSCs and differentiated MSCs. (**A**) RT-PCR analysis of the osteogenic-differentiation assay in terms of the osteogenic marker genes *Clo1a1*, *OPN*, and *OCN*. (**B**) RT-PCR analysis of the adipogenic-differentiation assay in terms of the adipogenic marker genes *Adipsin* and *PPARγ*. (**C**) RT-PCR analysis of chondrogenic-differentiation assay in terms of the chondrogenic marker genes *ACAN*, *Sox9*, and *Col2a1*.

Supplemental Figure 4

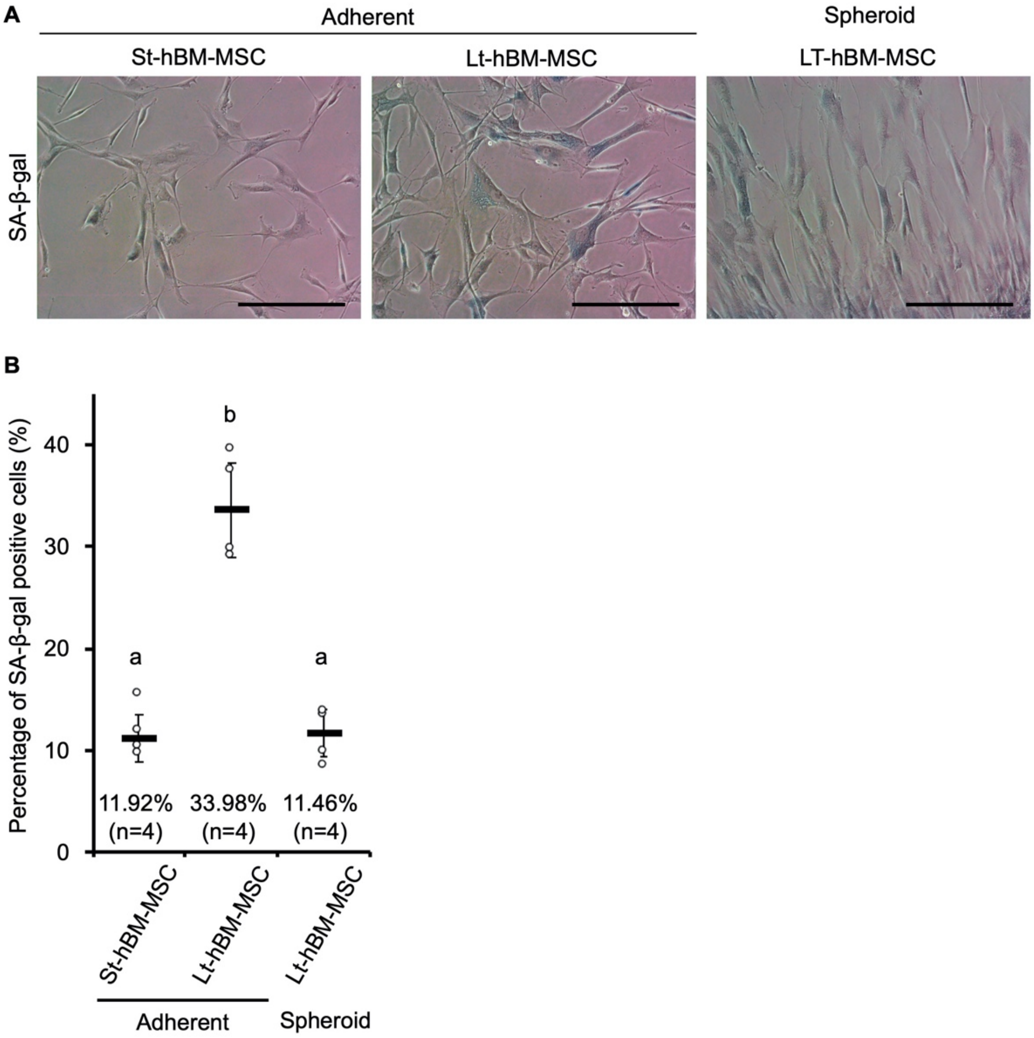

Supplemental Figure 4. Cell senescence analysis of hBM-MSC spheroids. (**A**) Phase contrasts of adherent St- and Lt-hBM-MSCs and migrated cells from re-attached spheroid stained by SA-β-gal. (**B**) Percentage of SA-β-gal positive cells. Graph bars: mean ± SD. *P < 0.05, Tukey's multiple-comparison test.

Supplemental Figure 5

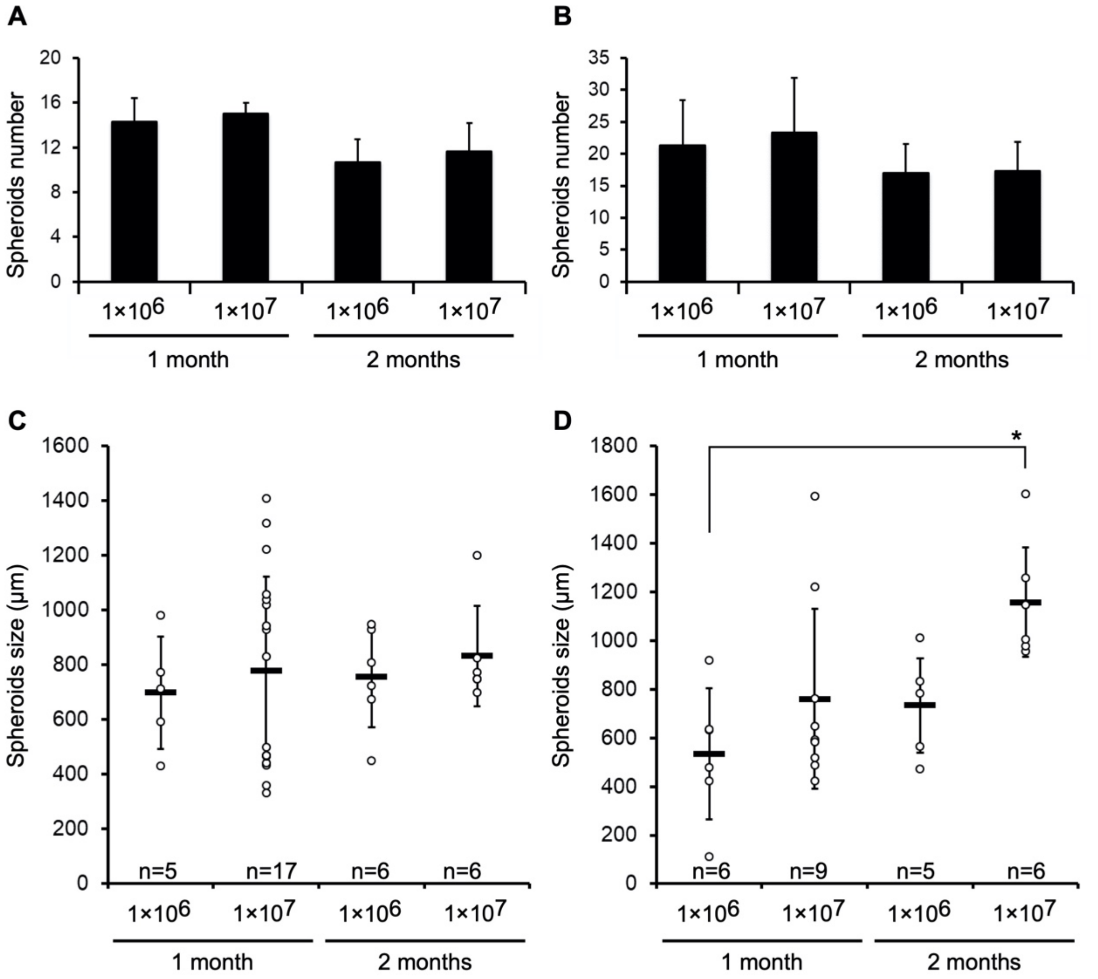

Supplemental Figure 5. Numbers and sizes of St-hBM-MSC and Lt-hBM-MSC spheroids. (**A**) Numbers of St-hBM-MSC spheroids cultured for 1 or 2 months. (**B**) Numbers of Lt-hBM-MSC-spheroids cultured for 1 or 2 months. (**C**) Sizes of St-hBM-MSC spheroids cultured for 1 or 2 months. (**D**) Sizes of Lt-hBM-MSC spheroids cultured for 1 or 2 months. Graph bars: mean ± SD. *P < 0.05, Tukey's multiple-comparison test.

Supplemental Figure 6

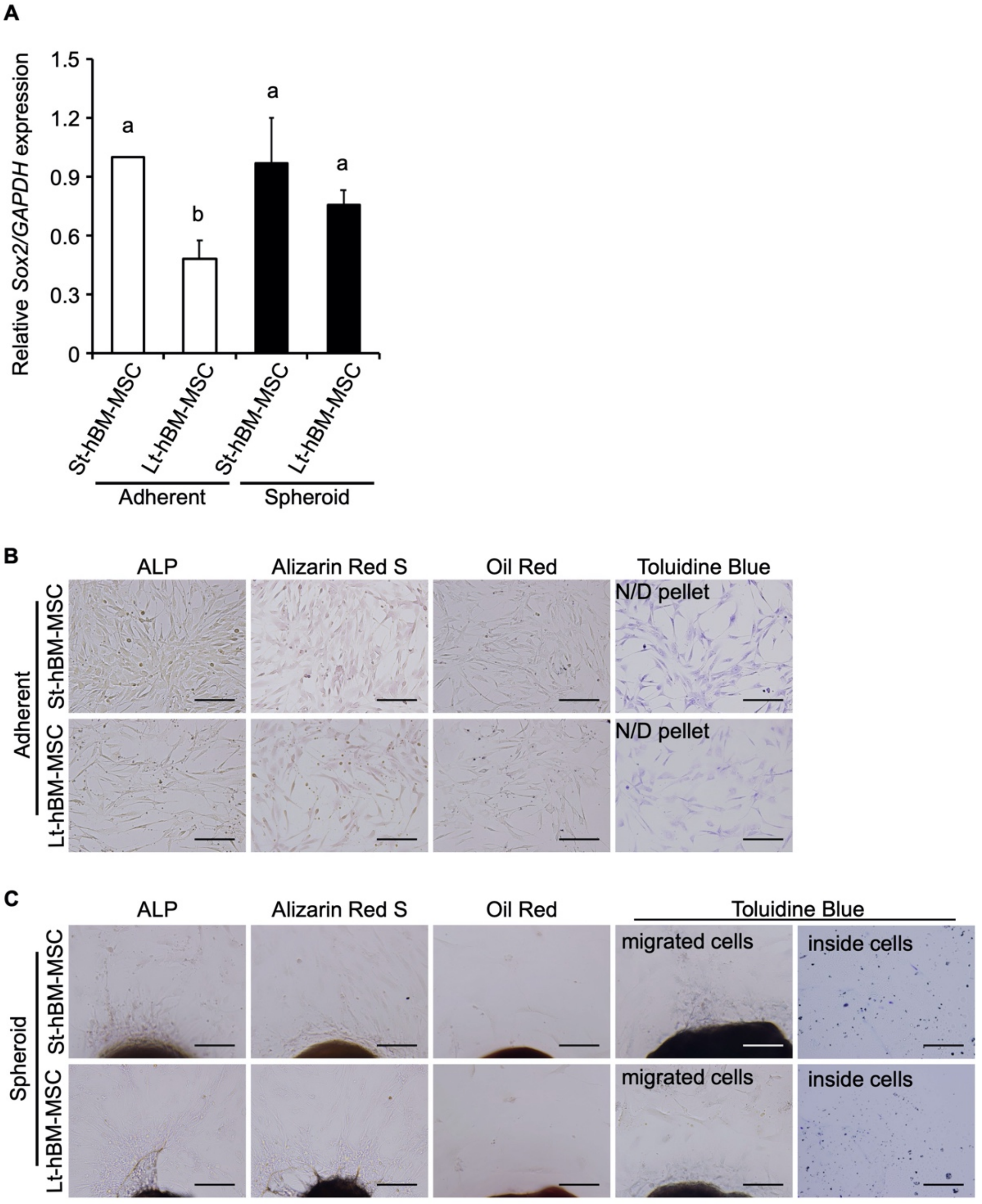

Supplemental Figure 6. Sox2 expression of hBM-MSC spheroids and negative control staining for differentiation assay. (**A**) Expression of *Sox2/GAPDH* was evaluated by real-time RT-PCR. (**B**) Staining with adherent cultured St-hBM-MSC and Lt-hBM-MSC under maintenance condition. Osteogenesis is indicated by ALP and Alizarin Red S stainings. Adipogenesis is indicated by neutral lipid vacuoles stained with

Oil red O. Chondrogenesis is indicated by toluidine blue staining. (**B**) Staining of migrated cells, from cultured St-hBM-MSC and Lt-hBM-MSC spheroids under maintenance condition. Osteogenesis is indicated by ALP staining and Alizarin Red S staining. Adipogenesis is indicated by neutral lipid vacuoles, which were stained with Oil red O. Chondrogenesis is indicated by toluidine blue staining. Left: cells migrated from spheroids. Right: inside of a spheroid. Scale bars: 200 μm.

Supplemental Figure 7

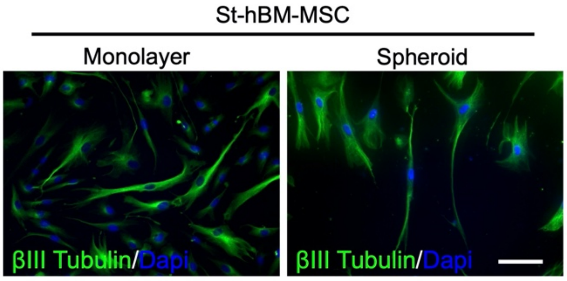

Supplemental Figure 7. Neurogenic potential of hBM-MSC spheroids. Immunocytochemistry of monolayered MSCs and MSC spheroids from St-hBM-MSCs after neural induction. Neuron marker: βIII tubulin. Nuclear stain: DAPI. Scale bars: 200 μm.
